# Inhibiting Arginine Methylation as a Tool to Investigate Cross-Talk with Methylation and Acetylation Post-Translational Modifications in a Glioblastoma Cell Line

**DOI:** 10.3390/proteomes6040044

**Published:** 2018-10-20

**Authors:** Sabrina Francesca Samuel, Alistair James Marsden, Srihari Deepak, Francisco Rivero, John Greenman, Pedro Beltran-Alvarez

**Affiliations:** 1 Biomedical Sciences, Faculty of Health Sciences, University of Hull, HU6 7RX Hull, UK; s.samuel@2017.hull.ac.uk (S.F.S.); a.marsden@2014.hull.ac.uk (A.J.M.); j.greenman@hull.ac.uk (J.G.); 2 Department of Neurosurgery, Hull Royal Infirmary, HU3 2JZ Hull, UK; Srihari.Deepak@hey.nhs.uk; 3 Hull York Medical School, Faculty of Health Sciences, University of Hull, HU6 7RX Hull, UK; francisco.rivero@hyms.ac.uk

**Keywords:** arginine methylation, cross-talk, glioblastoma, inhibitor, lysine acetylation

## Abstract

Glioblastomas (GBM) are the most common grade 4 brain tumours; patients have very poor prognosis with an average survival of 15 months after diagnosis. Novel research lines have begun to explore aberrant protein arginine methylation (ArgMe) as a possible therapeutic target in GBM and ArgMe inhibitors are currently in clinical trials. Enzymes known as protein arginine methyltransferases (PRMT1-9) can lead to mono- or di-ArgMe, and in the latter case symmetric or asymmetric dimethylation (SDMA and ADMA, respectively). Using the most common GBM cell line, we have profiled the expression of PRMTs, used ArgMe inhibitors as tools to investigate post-translational modifications cross-talk and measured the effect of ArgMe inhibitors on cell viability. We have identified novel SDMA events upon inhibition of ADMA in GBM cells and spheroids. We have observed cross-talk between ADMA and lysine acetylation in GBM cells and platelets. Treatment of GBM cells with furamidine, a PRMT1 inhibitor, reduces cell viability in 2D and 3D models. These data provide new molecular understanding of a disease with unmet clinical needs.

## 1. Introduction

Glioblastoma (GBM) is a very aggressive form of brain tumour with a universally poor prognosis. Clinical treatment options for GBM are mainly based on radio- and chemotherapy (principally temozolomide) regimens after surgical resection [1]. However, survival remains unacceptably low at an average of 15 months [2] and current research efforts are focused on combining temozolamide with other anticancer agents and on the development of new drugs to increase survival [3,4].

Arginine methylation (ArgMe) is a protein post-translational modification (PTM) specifically catalysed by enzymes known as protein arginine methyl transferases (PRMTs); the major PRMTs being PRMT1 and -5 [5,6,7]. PRMTs transfer methyl groups from S-adenosyl-L-methionine to produce monomethyl arginine. Additionally, Type I PRMTs, including PRMT1, -2, -3, -4, -6, and -8, lead to asymmetric dimethylation of arginine (ADMA), whilst Type II PRMTs including PRMT5 and -9 cause symmetrical dimethylation of target Arg residues (SDMA). PRMT7 is unique in that it produces monomethyl arginine only [8]. The three distinct ArgMe modifications (monomethylation, ADMA and SDMA) cause differential effects on protein functions and ultimately cell biology. Cell-permeable ArgMe inhibitors have been developed over the last decade and PRMT inhibition is currently being investigated as a possible therapeutic measure for the treatment of cancer in cell and animal models [9,10,11,12,13] and in clinical trials (identifiers NCT03573310, NCT02783300 and NCT03614728), including GBM. These studies have focused on targeting the major enzymes, PRMT1 and -5, in part due to the availability of very effective knock-down systems and specific inhibitors [14].

The present work addresses two knowledge gaps in the field. First, whether other members of the PRMT family, other than PRMT1 and -5, can be targeted in GBM. Second, molecular off-target effects of ArgMe inhibition are underexplored. In particular, the effects of inhibiting ArgMe on other PTMs have not been investigated in the setting of GBM. Within this context, our work uses ArgMe inhibitors as tools to identify PTM cross-talk in a commonly used GBM cell line.

## 2. Materials and Methods

### 2.1. Cell Culture and Drug Treatments

U87-MG cells were cultured in Dulbecco’s modified Eagle’s medium (Sigma, Kawasaki, Japan), supplemented with 10% foetal bovine serum. U87-MG spheroids were produced by culture on 1.5% agarose gel following published protocols [15]. Cells were treated with the following PRMT inhibitors: AdOx, AMI-1, GSK591 (all from Sigma), MS023 and furamidine (both from Tocris Bioscience, Bristol, UK).

### 2.2. Platelet Isolation

Platelets were isolated from whole blood of healthy donors, as previously described [16], resuspended in modified Tyrode’s buffer (20 mM HEPES, 134 mM NaCl, 2 mM KCl, 0.34 mM Na_2_HPO_4_, 12 mM NaHCO_3_, 1 mM MgCl_2_, 5.6 mM Glucose, pH 7.3), treated with furamidine for 4 h at 37 °C and then lysed in Laemmli buffer. All protocols were completed in accordance with the University of Hull and Hull York Medical School (HYMS) ethical guidelines. Work with platelets was approved by the HYMS ethics committee and was completed under the project ‘1501: the study of platelet activation signalling and metabolism’.

### 2.3. Western Blot

U87-MG cells and spheroids were seeded onto 6-well and 96-well plates, respectively, at an appropriate density and treated with PRMT inhibitors for 48–144 h. Cells were harvested and lysed in 5% SDS. Proteins (20–100 μg) were separated by SDS-PAGE in 12% gels and transferred to nitrocellulose membranes. Membranes were blocked in 5% milk powder in TBST (50 mM Tris-HCl, 150 mM NaCl, 0.05% Tween-20, pH = 7.4) for 1 h and incubated in primary antibody (diluted in 5% milk in TBST) overnight at 4 °C. Following three washes with TBST, membranes were incubated with HRP-conjugated secondary antibody (Dako, Beijing, China) for 1 h at room temperature, washed and signals visualised by incubation with HRP substrate (Millipore, Burlington, MA, USA). All PRMT antibodies were from Abcam. SDMA, ADMA and Lys acetylation antibodies were from Cell Signaling Technologies (Danvers, MA, USA). Representative blots of at least two independent experiments are shown. Glyceraldehyde 3-phosphate dehydrogenase (GAPDH) was used as a control for total protein loading.

### 2.4. Cell Viability Assays

Cells were seeded onto 96-well plates at an appropriate density and treated with either a serial dilution (0–100 µM) or 100 µM PRMT inhibitors. Cell viability was determined by MTS assay according to instructions of the manufacturer (Abcam, Cambridge, UK). Absorbance was measured using a microplate reader. A minimum of 3 biological replicates are represented by averaged values ± SD. Statistical significance of differences between paired samples were determined using Student’s *t*-test.

## 3. Results

### 3.1. U87-MG Cells Express a Wide Range of PRMTs and the Use of PRMT Inhibitors Decreases ArgMe Profiles

**Figure 1:**
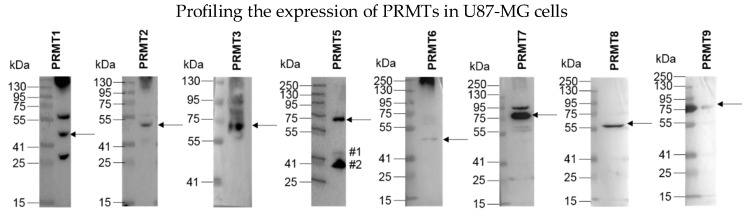
Detection of PRMT expression in U87-MG cells. Expected molecular weights (arrows), from left to right: PRMT1—42 kDa, PRMT2—46 kDa, PRMT3—60 kDa, PRMT5—73 kDa, PRMT6—42 kDa, PRMT7—78 kDa, PRMT8—45 kDa, PRMT9—94 kDa. Full membranes representative of at least two independent experiments are shown. The PRMT5 membrane was subsequently blotted with anti-PRMT1 and anti-GAPDH antibodies (bands labelled as #1 and #2, respectively). Additional bands recognized by the PRMT1 antibody could correspond to PRMT1 isoforms, of which at least eight are known in the 36–43 kDa range [18].

To gain an understanding of the scope of ArgMe in U87-MG cells, we first profiled the expression of PRMT1-9 using Western blot and antibodies specific to each PRMT. We could detect expression of all PRMTs, except for PRMT4/CARM1 (Figure 1). To directly characterise ArgMe in U87-MG cells, we used a panel of ArgMe inhibitors specific for given PRMTs. We incubated U87-MG cells with general, Type I PRMT and PRMT5 inhibitors (Table 1) for 48–72 h.

**Figure 2:**
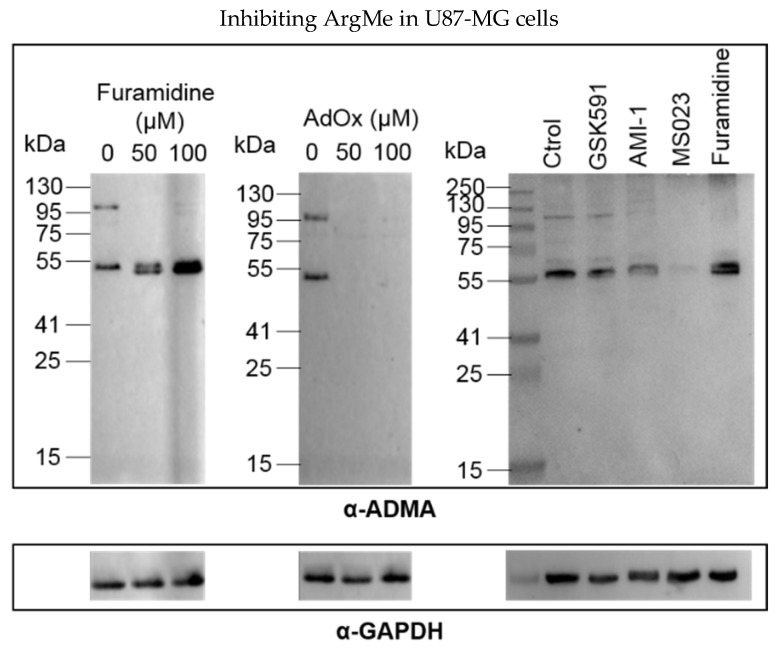
Detection and inhibition of ADMA in U87-MG cells. Left and central panels, dose-response of ADMA inhibition by furamidine and AdOx, respectively. Right panel, ADMA profiles after incubation of U87-MG cells with 100 µM of the indicated inhibitor for 48 h. ADMA was detected using an α-ADMA antibody (#13522 Cell Signaling Technologies, Danvers, MA, USA). For each panel, GAPDH is shown below as a total protein loading control.

Incubation of U87-MG cells with furamidine led to loss of ADMA of a protein next to the 95 kDa marker, while the intensity of the lower band at 55 kDa increased (Figure 2, left panel). The effect of furamidine was specific because incubation of cells with the general ArgMe inhibitor AdoX suppressed ArgMe activity (Figure 2, central panel). Intriguingly, incubation of cells with MS023 (and, to a lesser extent, AMI-1) recapitulated the loss of ADMA at both the 55 and 95 kDa marks (Figure 2, right panel). Given that furamidine is specific to PRMT1, while MS023 is a broad Type I PRMT inhibitor [17], we argue that the increase in ADMA at 55 kDa may well be due to other Type I PRMT activity upon specific inhibition of PRMT1 by furamidine. As expected, GSK591 did not inhibit ADMA. Together, these data identify other potential PRMTs that can be of interest in GBM research out of the PRMT1/5 paradigm and suggest diverse ArgMe activity in U87-MG cells, which can be efficiently inhibited using small molecules.

### 3.2. Using PRMT Inhibitors as a Tool to Investigate ADMA‒SDMA and ADMA‒Lys Acetylation Cross-Talks

Having established a cell model amenable for the study of ArgMe as well as the effectiveness of PRMT inhibitors in our system, we set out to investigate ArgMe cross-talks. Based on previous data on the cardiac arginine methylome [19], we hypothesised that ArgMe would cross-talk with other ArgMe events and with Lys modifications. To test this hypothesis, we incubated U87-MG cells with specific PRMT inhibitors and searched for new ArgMe and Lys acetylation and methylation marks.

**Figure 3:**
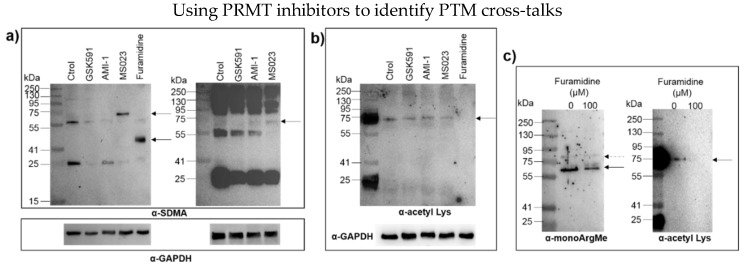
ADMA cross-talks with SDMA and Lys acetylation. (**a**) Identification of novel SDMA events (labelled with arrows) upon inhibition of U87-MG cells with MS023 and furamidine for 48 h (2D models, left) and with MS023 for 96 h (3D models, right). The α-SDMA antibody used was #13222 from Cell Signaling Technologies; (**b**) inhibition of Lys acetylation of a protein at ca. 75 kDa (labelled with an arrow) upon treatment of U87-MG cells with furamidine; (**c**) in platelets, furamidine led to reduced monoArgMe in a specific manner (solid arrow) while other ArgMe marks were not affected (dashed arrow), left panel. The α-monoArgMe antibody used was #8711 from Cell Signaling Technologies. Lys acetylation of a protein at ca. 75 kDa was lost upon treatment of platelets with furamidine (right panel). The α-Lys acetylation antibody used in panels b and c was #9441 from Cell Signaling Technologies. In all cases, the corresponding PRMT inhibitor was added at a concentration of 100 μM.

Upon inhibiting Type I PRMTs with MS023 and furamidine, we observed new protein bands revealed by SDMA antibodies at approximate molecular weights of 75 kDa and 45 kDa, respectively (Figure 3a, left panel). These observations indicate that ADMA inhibition facilitates SDMA deposition in trans, although the identity of the different proteins is unknown at the moment. MS023 is known to target Type I PRMTs including PRMT1, -3, -6 and -8 [20], while furamidine is thought to be specific for PRMT1 [17]. This difference in specificity could partially explain why distinct and new SDMA bands were detected in each case. While AMI-1 inhibits mainly Type I PRMTs, it has also been shown to inhibit PRMT5 [21], which likely explains why no new SDMA bands were detected upon incubation of U87-MG cells with AMI-1. ADMA–SDMA cross-talk did not seem to be bidirectional because incubation of cells with GSK591, which targets the Type II PRMT5 and inhibits SDMA, did not lead to new ADMA events (Figure 2, right panel).

Cross-talk between ADMA and SDMA was observed consistently in 2D cell culture models. To test if this interplay was conserved in 3D models, we grew U87-MG spheroids and treated them with PRMT inhibitors. We anticipated new SDMA bands at 75 and 45 kDa in spheroids incubated with MS023 and furamidine, respectively. Upon incubation of spheroids with MS023, we observed a novel (though weak) SDMA band at ca. 75 kDa (Figure 3a, right panel, indicated by an arrow), with concomitant reduction of the intensity of the protein band at 55 kDa. Although we did not detect extra SDMA bands when spheroids were incubated with furamidine (not shown), overall our observations suggest similar PRMT activities in 2D and 3D culture models of U87-MG cells.

We then focused our attention on ArgMe-Lys PTMs. We could not detect cross-talk between ArgMe and Lys methylation (not shown). On the other hand, inhibition of PRMT1 with furamidine led to decreased Lys acetylation profiles, including loss of Lys acetylation of a protein at ca. 75 kDa (Figure 3b). This indicates either non-specific inhibition of Lys acetyltransferases by furamidine, which has not previously been described, or cross-talk between PRMT1-catalysed ArgMe and Lys acetylation in U87-MG cells. To obtain independent evidence supporting ArgMe-Lys acetylation cross-talk, we then used furamidine in a different cell model. Being anucleated cells, platelets are a useful model to study PTMs because of their very limited capability of protein synthesis [22]. We incubated platelets with furamidine for 4 h and observed a significant and specific reduction in ArgMe (Figure 3c, left panel) with concomitant loss of Lys acetylation of a protein at ca. 75 kDa (Figure 3c, right panel), which is remarkably consistent with our results using U87-MG cells.

### 3.3. Inhibition of ArgMe Leads to Decreased U87-MG Cell Viability in 2D and 3D Models

**Figure 4:**
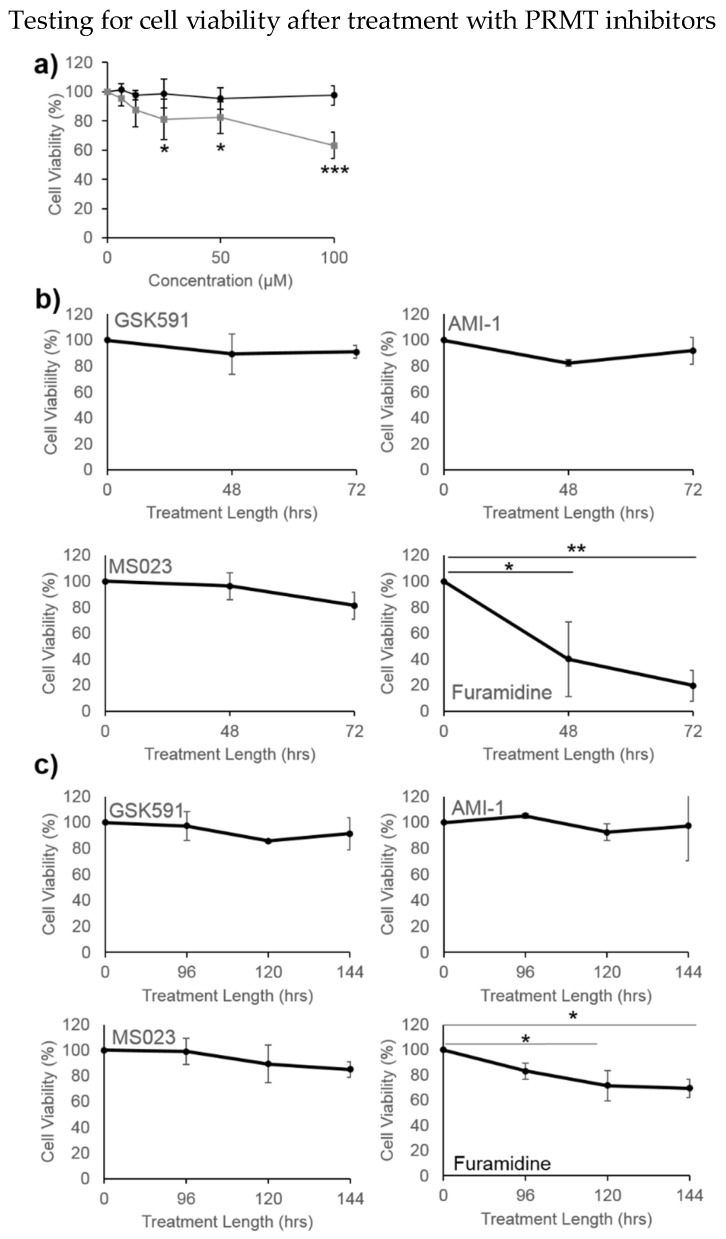
U87-MG cell viability upon incubation with PRMT inhibitors. (**a**) Dose-response after treatment with furamidine (grey squares) and GSK-591 (black circles) for 48 h in 2D cell culture; (**b**) effect of 100 µM GSK591, AMI-1, MS023 or furamidine on cells grown in 2D cultures; (**c**) effect of 100 µM GSK591, AMI-1, MS023 or furamidine on cells grown in spheroids. * *p* < 0.05, ** *p* < 0.01, *** *p* < 0.001 with respect to no treatment or initial time point.

To explore the significance of ArgMe in U87-MG cells, we asked what effect PRMT inhibitors have on U87-MG cell viability. We first incubated U87-MG cells with several concentrations of furamidine and found a dose-dependent reduction in cell viability (Figure 4a). We incubated cells with our panel of ArgMe inhibitors under standard 2D cell culture conditions (Figure 4b) as well as spheroids (Figure 4c). We observed decreased cell viability when cells were incubated with furamidine in both the 2D and 3D models. This effect was specific, because incubation of cells with GSK591, AMI-1 or MS023 did not statistically affect U87-MG viability.

## 4. Discussion

There is a clear need for novel and more effective therapeutic approaches for the treatment of GBM. Our timely work contributes to the molecular understanding of GBM by describing the PRMTs that are expressed in the common GBM cell line U87-MG, by identifying PTM cross-talk upon treatment of U87-MG cells with ArgMe inhibitors and by analysing the effect of ArgMe inhibitors on U87-MG cell viability.

We have observed off-target PTM events as a consequence of ArgMe inhibition, including new SDMA bands and loss of Lys acetylation. ADMA‒SDMA cross-talk seemed to be conserved in MS023-treated 2D and 3D U87-MG models, although the intensity of the proposed, new SDMA band was lower in spheroids. This may be due to reduced diffusion of ArgMe inhibitors into spheroids, which could also explain why the effects of furamidine on cell viability were decreased in 3D compared to 2D U87-MG models. We expected similar effects on PTM cross-talks when incubating cells with MS023 and furamidine as both have recently been described as ADMA inhibitors [17,20], but that was not the case. It is unlikely that these differing results are due to the detection of kinetic intermediates because of the relatively high inhibitor concentrations used (100 µM) and length of exposure (48–72 h). We favour the hypothesis that PRMT1 inhibition by furamidine enables other Type I PRMT activity (as suggested by Figure 2) which, in turn, could lead to cross-talk with Type II PRMTs and Lys acetyltransferases. It is known, for example, that several PRMTs and the ubiquitous Lys acetyltransferase p300 are modified by ArgMe [23,24,25].

This study has limitations related to its small size. First, our observations open an intriguing question with respect to the effects of ArgMe inhibitors on cell viability (described here for U87-MG cells and by other groups in the wider cancer field), that is, is the reduced viability of cells treated with ArgMe inhibitors a direct consequence of ArgMe inhibition, or rather the effect of other methylation and acetylation marks on unknown proteins subsequent to ArgMe inhibition (or both)? We anticipate that the impact of using ArgMe inhibitors on other PTMs and associated disease outcomes will shortly gain much visibility, bearing in mind the launch of clinical trials to test PRMT inhibitors in the setting of cancer (including GBM cohorts). The molecular mechanisms of any therapeutic effects will need investigation and our work suggests that PTM interplays can play a role. Second, the present work relies on antibody specificity to draw the conclusion that ADMA cross-talks with SDMA and Lys acetylation. Although the α-ADMA, α-SDMA and α-Lys acetylation antibodies that we have used are state-of-the art in the field [26,27,28,29,30,31,32,33,34,35,36,37,38,39,40,41,42,43,44,45,46], mass spectrometry-based proteomics approaches would complement our experiments and enable the identification of the proteins and mechanisms involved in PTM cross-talks. In particular, approaches to labelling Arg residues modified by methylation with ^14^C and ^13^CD_3_ have been developed [47,48], and would prove very useful to identify the specific proteins undergoing methylation in further investigations of ADMA–SDMA cross-talk. In this respect, the development of tools for studying PTM cross-talk is critical and this Special Issue of *Proteomes* will undoubtedly contribute to it.

## Figures and Tables

**Table 1:** Protein arginine methyltransferases (PRMT) inhibitors used in this work and their specificities (see text for references).

Inhibitor	Specificity
AdOx	Broad
AMI-1	Broad
MS023	PRMT1, -3, -6
Furamidine	PRMT1
GSK591	PRMT5
